# MINIMALLY INVASIVE HALLUX VALGUS SURGERY: LESSONS FROM 1,217 FEET OVER EIGHT YEARS AT A SINGLE CENTER

**DOI:** 10.1590/1413-785220263406e309112

**Published:** 2026-11-09

**Authors:** Gabriel Ferraz Ferreira, Juliana Vilela Lacerda, Laryssa Augustinho dos Santos, Fernando Len, Giovane Aloise Cruz, Miguel Viana Pereira

**Affiliations:** 1 Departamento de Ortopedia e Traumatologia, Prevent Senior, Sao Paulo, SP, Brazil.; 2 Instituto Vita, Sao Paulo, SP, Brazil.; 3 Universidade Estadual de Campinas (UNICAMP), Faculdade de Medicina, Departamento de Ortopedia, Reumatologia e Traumatologia, Campinas, SP, Brazil.

**Keywords:** Hallux Valgus, Minimally Invasive Surgical Procedures, Osteotomy, Orthopedic Procedures, Hallux Valgus, Procedimentos Cirúrgicos Minimamente Invasivos, Osteotomia, Procedimentos Ortopédicos

## Abstract

**Objective:**

To document the eight-year institutional evolution of minimally invasive hallux valgus surgery at a single high-volume center, describing the sequential transitions between operative techniques and the clinical and biomechanical rationale that motivated each change.

**Methods:**

Retrospective analysis of prospectively collected data from January 2018 through December 2025. All feet undergoing primary minimally invasive hallux valgus surgery performed by the same team were included. Annual distributions of technique: Reverdin-Isham (RI), Minimally Invasive Chevron-Akin (MICA, 3rd-generation), metaphyseal extra-articular transverse and Akin osteotomy (META, 4th-generation), and combined percutaneous exostectomy-Akin (EA), bilateral procedure rates, and surgical volume were analyzed.

**Results:**

A total of 1,217 feet were operated on during the study period. RI predominated in 2018-2019 (n=181), was supplanted by MICA from 2020-2021, and META became the primary institutional procedure from April 2022 onward, accounting for 51.7% (n=629) of all procedures by study end. The combined EA approach declined from 26.0% in 2019 to 0% in 2023 (p<0.001). Bilateral simultaneous procedures decreased significantly from 45.2% (2024) to 34.0% (2025) (p=0.020), driven by evidence of elevated fracture risk in bilateral cases.

**Conclusion:**

This study documents a continuous, evidence-driven evolution of minimally invasive hallux valgus surgery over eight years at a high-volume, single-team center. The META technique has emerged as the institutional standard due to its biomechanical safety and pedagogical advantages. These findings provide a practical framework for centers undergoing similar technical transitions. *Level of Evidence IV; Retrospective Observational Study.*

## RESUMO

**Objetivo:**

Documentar a evolução institucional, ao longo de oito anos, da cirurgia minimamente invasiva do hálux valgo em um único centro de alto volume, descrevendo as transições sequenciais entre as técnicas operatórias e os fundamentos clínicos e biomecânicos que motivaram cada mudança.

**Métodos:**

Análise retrospectiva de dados coletados prospectivamente entre janeiro de 2018 e dezembro de 2025. Foram incluídos todos os pés submetidos à cirurgia minimamente invasiva primária do hálux valgo, realizada pela mesma equipe. Foram analisadas as distribuições anuais das técnicas: Reverdin-Isham (RI), Chevron-Akin minimamente invasiva (MICA, 3ª geração), osteotomia metafisária transversa extra-articular associada à osteotomia de Akin (META, 4ª geração) e exostectomia percutânea combinada à osteotomia de Akin (EA), bem como as taxas de procedimentos bilaterais e o volume cirúrgico.

**Resultados:**

Um total de 1.217 pés foi operado durante o período do estudo. A técnica RI predominou em 2018-2019 (n=181), foi substituída pela MICA a partir de 2020-2021, e a META tornou-se o principal procedimento institucional a partir de abril de 2022, correspondendo a 51,7% (n=629) de todos os procedimentos ao final do estudo. A abordagem combinada EA diminuiu de 26,0% em 2019 para 0% em 2023 (p<0,001). Os procedimentos bilaterais simultâneos diminuíram significativamente de 45,2% (2024) para 34,0% (2025) (p=0,020), em razão de evidências de maior risco de fratura nos casos bilaterais.

**Conclusão:**

Este estudo documenta uma evolução contínua, orientada por evidências, da cirurgia minimamente invasiva do hálux valgo ao longo de oito anos em um centro de alto volume, com atuação de uma única equipe. A técnica META consolidou-se como o padrão institucional em razão de sua segurança biomecânica e de suas vantagens no ensino da técnica cirúrgica. Esses achados fornecem um referencial prático para centros que estejam passando por transições técnicas semelhantes. *Nível de Evidência IV; Estudo Observacional Retrospectivo.*

## INTRODUCTION

Hallux valgus is among the most prevalent forefoot deformities requiring surgical intervention, with an estimated global prevalence approaching 23% of adults.^1^ Furthermore, the volume of scientific publications on hallux valgus has increased substantially in recent years, with continued growth expected.^2^

Its surgical management has historically relied on open osteotomy techniques; however, minimally invasive approaches have undergone a significant renaissance over the past two decades, driven by advances in dedicated burrs, fluoroscopic guidance, and internal fixation hardware that permit reproducible correction through incisions of only a few millimeters.^2-7^

The evolution of minimally invasive hallux valgus surgery can be understood as a succession of technical generations, each addressing the limitations of its predecessor.^8^

First-generation techniques, including the Reverdin-Isham (RI) osteotomy, offered technical simplicity and low implant burden but relied on dressing-based stabilization without rigid internal fixation.^9-12^

Second-generation minimally invasive hallux valgus surgery, typified by the Bösch technique, used a percutaneous subcapital transverse osteotomy stabilized with an intramedullary Kirschner wire under fluoroscopic guidance.^13,14^

Third-generation techniques, most prominently the Minimally Invasive Chevron-Akin (MICA) procedure as refined by Redfern and Vernois, introduced stable dual-screw fixation with extreme lateral translation of the metatarsal head, demonstrating outcomes comparable to open Scarf-Akin procedures.^7,15,16^

More recently, the fourth-generation Metaphyseal Extra-Articular Transverse Akin (META) osteotomy was introduced, substituting a straight transverse for the chevron-shaped cut at the distal metaphysis.^17^ This modification eliminates the risk of creating a long lateral wing without adequate cortical purchase, a recognized pitfall of the chevron configuration, and offers a shallower learning curve for trainees.

Despite the expanding literature on individual techniques, longitudinal data documenting the institutional rationale and timing of each transition within a stable surgical team are scarce.

The present study reports our eight-year experience (2018-2025) at a single high-volume center in São Paulo, Brazil, capturing the sequential adoption of minimally invasive techniques and the observations that drove each change.

## METHODS

### Study Design

This was a retrospective observational study analyzing the evolution of minimally invasive hallux valgus techniques and surgical volume at a single center. The study was conducted at The study was conducted at Instituto Prevent Senior, São Paulo, Brazil, where all clinical evaluations and surgical procedures were performed following standardized institutional protocols.

### Ethical Approval

The study was approved by the local ethics committee and conducted in accordance with the Declaration of Helsinki and Good Clinical Practice guidelines.

### Participants

All consecutive feet with primary symptomatic hallux valgus treated at our institution between January 1, 2018, and December 31, 2025, were eligible for inclusion if they underwent minimally invasive correction using one of the techniques adopted during the study period: first-generation Reverdin-Isham osteotomy, third-generation MICA, fourth-generation META, or the combined percutaneous exostectomy-Akin procedure. All operations were performed by the same surgical team in a day-hospital setting. Revision cases and feet with incomplete institutional records were excluded. A minimum of six months of failed conservative treatment was required before surgical indication.

### Surgical Techniques

- Reverdin-Isham (RI) osteotomy. A first-generation subcapital percutaneous technique involving a subchondral medial-closing osteotomy and concomitant Akin osteotomy of the proximal phalanx, without internal fixation.^18^ Correction is maintained entirely by dressing immobilization.
- Minimally Invasive Chevron-Akin (MICA, 3rd-generation). An extracapsular chevron (V-shaped) osteotomy at the distal metaphysis of the first metatarsal using a 2.0 mm Shannon burr, as described by Redfern and Vernois.^6,7^ After extreme lateral translation of the metatarsal head, correcting varus, pronation, and sagittal imbalance under fluoroscopic guidance, the osteotomy is fixed with two beveled cannulated screws. Concomitant Akin osteotomy of the proximal phalanx was performed in all cases.^19,20^
- META osteotomy (4th-generation). The META technique employs a straight transverse osteotomy at the distal metaphysis of the first metatarsal using the same Shannon burr and dual-screw fixation principle as MICA.^17,21^
- Combined percutaneous exostectomy and Akin osteotomy (EA). Employed for very mild hallux valgus, involving medial eminence percutaneous excision and proximal phalangeal closing wedge osteotomy without fixation (Akin osteotomy).

### Postoperative Protocol

Immediate full weight-bearing was permitted in all cases using rigid-sole orthopaedic footwear. Perioperative analgesia followed a uniform institutional protocol consisting of ankle or Mayo block with ropivacaine.^22,23^ Postoperative wound management differed according to technique.

For patients treated with the Reverdin-Isham osteotomy, surgical dressings were changed weekly by the operating team for six weeks, with orthopaedic footwear maintained throughout this period. For patients treated with MICA or META, weekly dressing changes were performed by the surgical team during the first four weeks; following the final dressing change, patients were instructed to initiate active mobilization of the hallux to reduce the risk of postoperative stiffness. Transition to conventional footwear was authorized at six weeks contingent on satisfactory radiographic osseous healing.

### Data Collection

Patient data were recorded using anonymized numerical identifiers and managed through Research Electronic Data Capture (REDCap). REDCap is a secure, web-based software platform designed to support data capture for research studies, providing 1) an intuitive interface for validated data capture; 2) audit trails for tracking data manipulation and export procedures; 3) automated export procedures for seamless data downloads to common statistical packages, and 4) procedures for data integration and interoperability with external sources.^24^

### Statistical Analysis

Descriptive statistics included absolute counts, proportions, and annual totals. Differences in bilateral procedure rates between selected years and in aggregate exostectomy-Akin proportions between selected periods were evaluated using Pearson chi-square tests with continuity correction. Significance was set at p<0.05. All analyses were conducted using R version 4.4.0.^25^

## RESULTS

### Surgical Volume

Between January 2018 and December 2025, a total of 1,217 feet underwent minimally invasive surgery for hallux valgus at our institution (Figure 1).

**Figure 1:**
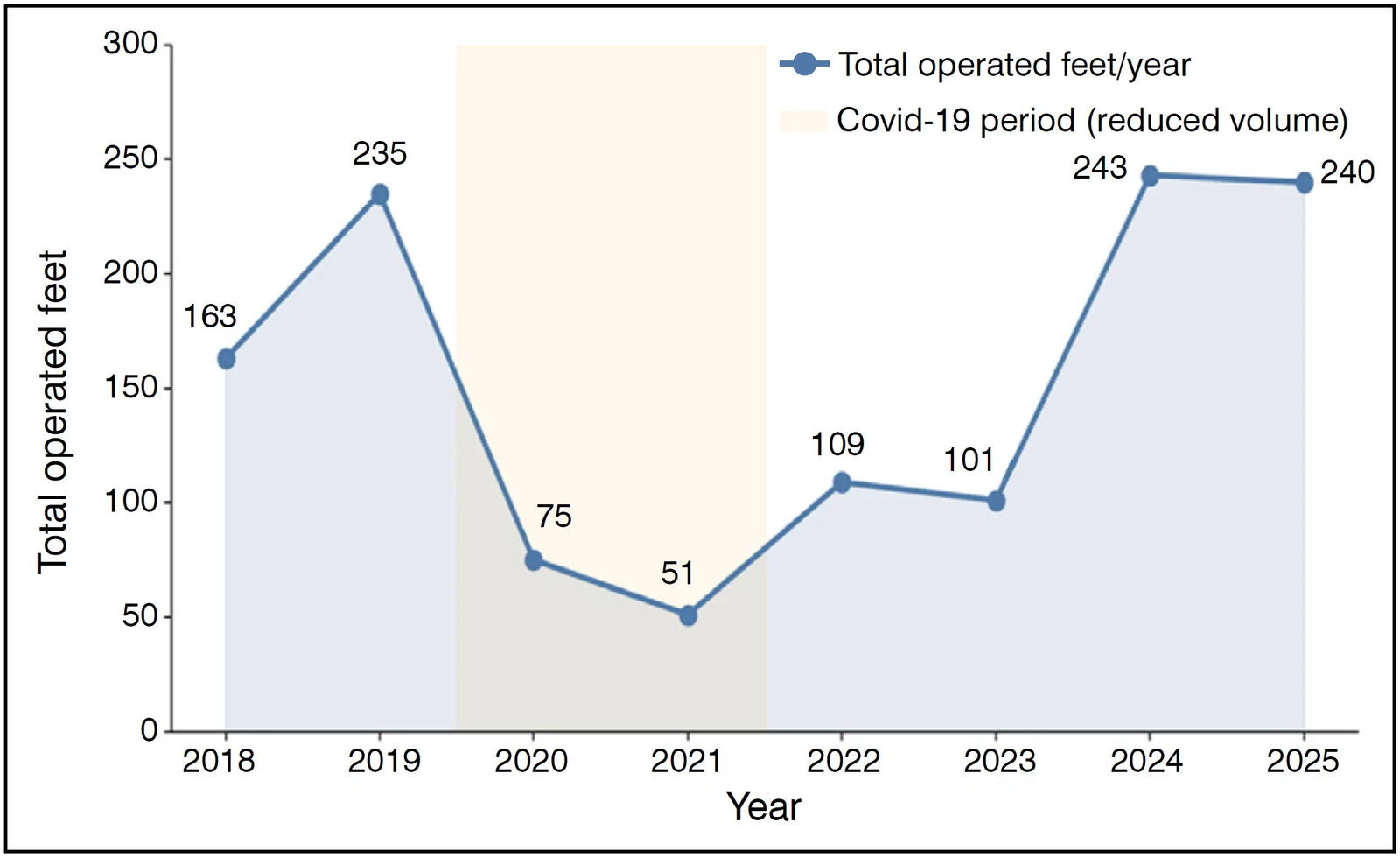
Annual surgical volume of minimally invasive hallux valgus procedures (2018-2025).

Annual volume ranged from 51 feet in 2021, the nadir imposed by COVID-19 pandemic restrictions on elective surgery, to 243 feet in 2024. Following the recovery of full institutional activity in 2022, annual volume exceeded 100 feet in each subsequent year, with 240 feet operated in 2025. The consistent high volume across the study period reflects the center's role as a specialized referral unit supported by a stable, dedicated surgeon team.

### Technique Distribution

The annual distribution of operative techniques is illustrated in Figure 2.

**Figure 2:**
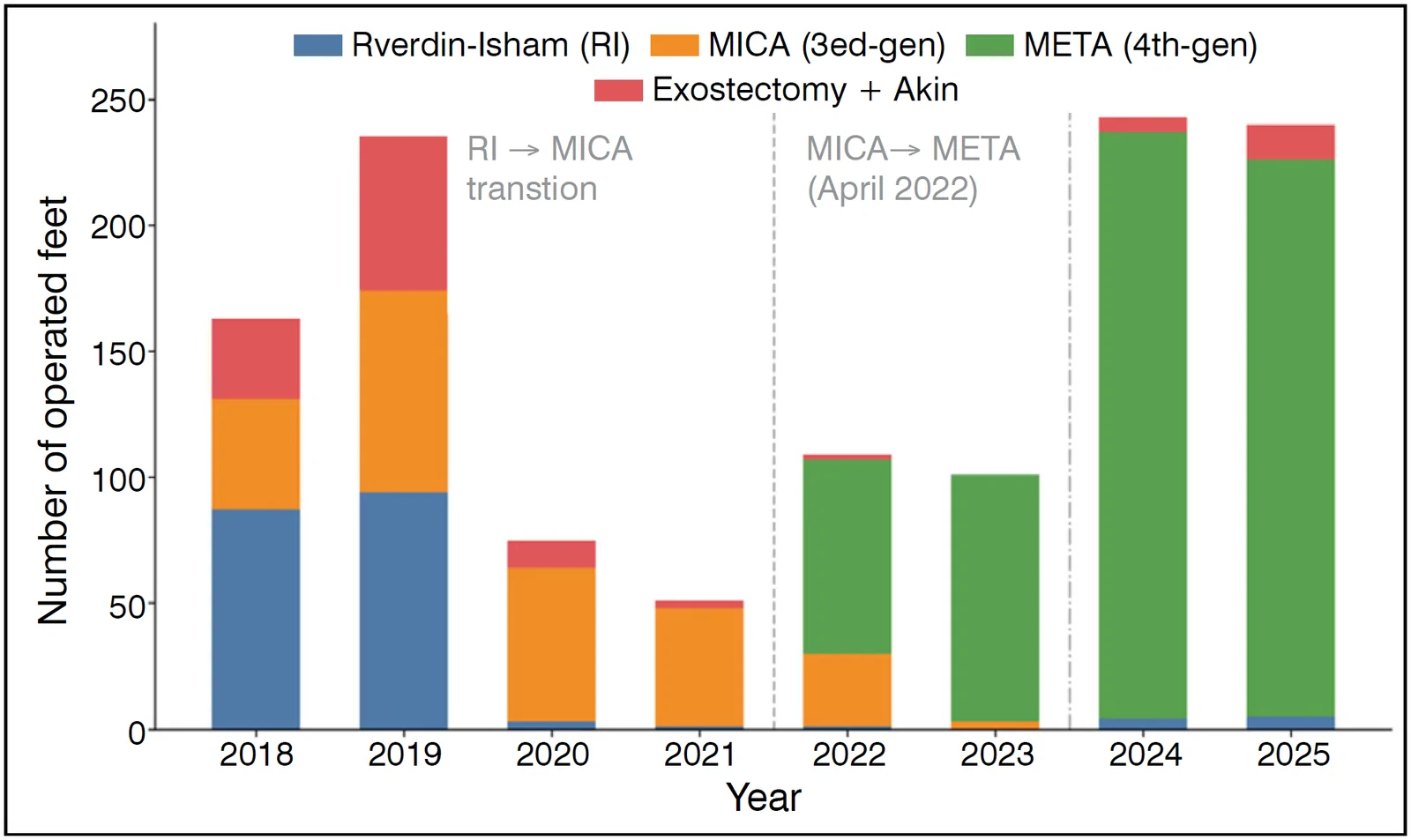
Annual distribution of minimally invasive hallux valgus techniques (2018-2025).

The RI osteotomy predominated in 2018 (n=87; 53.4%) and 2019 (n=94; 40.0%). Transition to MICA began during 2019. MICA became the dominant technique in 2020 (n=61; 81.3%) and 2021 (n=47; 92.2%), with near-complete abandonment of RI. In April 2022, META became the primary institutional procedure.

By 2023, META accounted for 97.0% of procedures (n=98/101), a proportion maintained in 2024 (95.9%; n=233/243) and 2025 (92.1%; n=221/240). Only a few RI cases were performed from 2024 onward (n=4 in 2024 and n=5 in 2025).

### Exostectomy-Akin (EA) for Mild Deformity

The combined percutaneous EA procedure was performed in 129 feet (10.6% of the total sample), with highest use in 2019 (n=61; 26.0%). Its use declined thereafter, reaching 5.9% in 2021, 1.8% in 2022, and 0% in 2023, before limited reintroduction in selected cases in 2024 (n=6; 2.5%) and 2025 (n=14; 5.8%). The difference in overall proportions between 2018-2019 and 2022-2023 was significant (chi-square, p<0.001).

### Bilateral Simultaneous Procedures

Bilateral surgery was performed in 37.4% of cases in 2018 and peaked at 45.2% in 2024, reflecting a growing institutional preference for addressing both feet in a single anesthetic episode. A significant decline was observed in 2025 (34.0% vs. 45.2% in 2024; chi-square p=0.020). Bilateral procedure proportions are shown in Figure 3.

**Figure 3:**
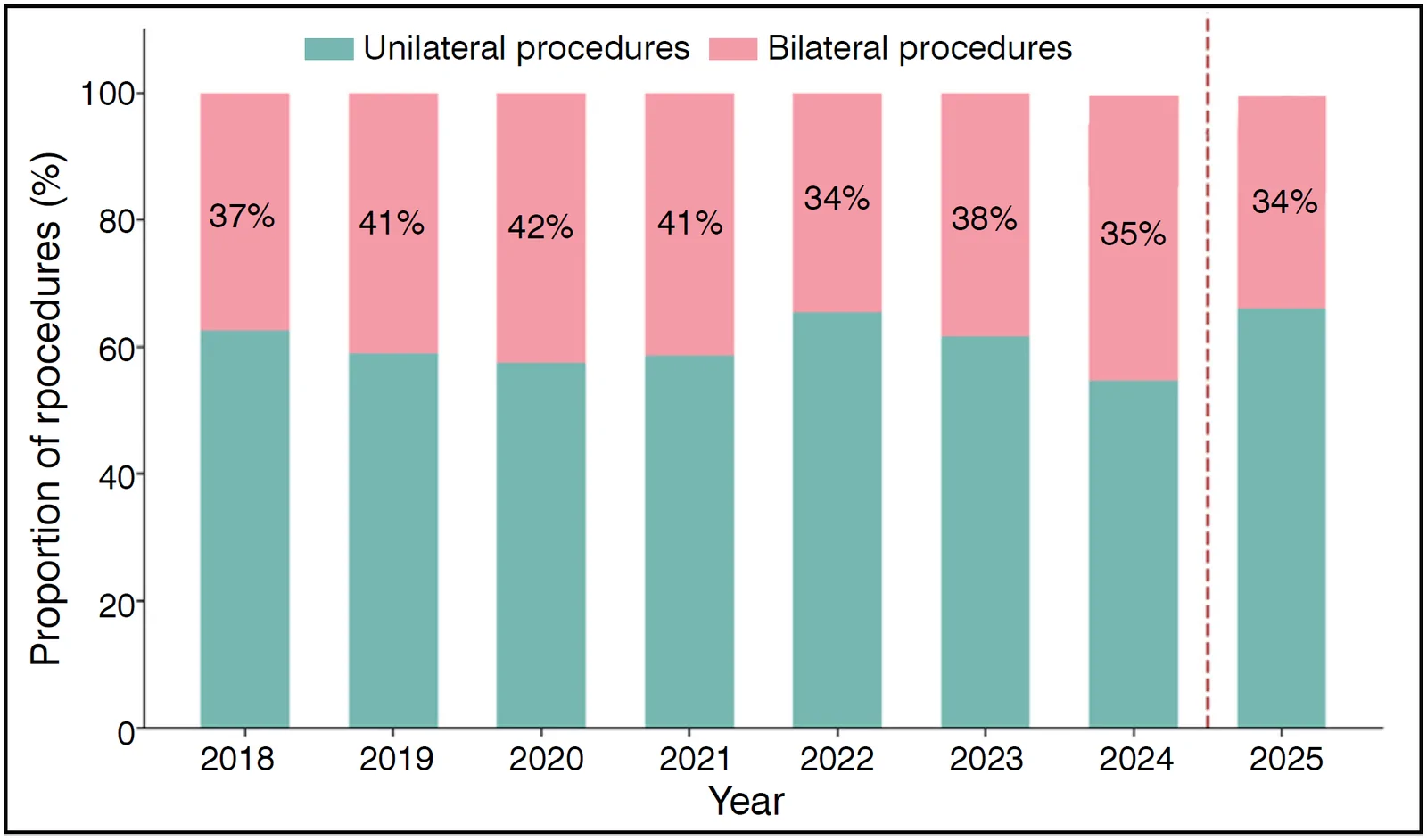
Proportion of unilateral versus simultaneous bilateral minimally invasive hallux valgus procedures by year (2018-2025).

## DISCUSSION

The present study documents an eight-year trajectory of minimally invasive hallux valgus surgery at a single high-volume center, capturing the sequential transitions between operative techniques and the clinical and biomechanical evidence that motivated each change. To our knowledge, this represents one of the longest continuously documented single-team series of minimally invasive hallux valgus surgery in the literature, comprising 1,217 feet operated on across eight consecutive years by the same surgical partnership.

The initial institutional predominance of the RI osteotomy in 2018-2019 is consistent with the historical learning trajectory of percutaneous surgery programs internationally, in which first-generation techniques often served as accessible training platforms.^18^

In our practice, however, utilization of the RI technique declined progressively and was largely discontinued over time. This shift reflected a combination of factors observed during follow-up, including postoperative digital stiffness and a higher-than-acceptable rate of deformity recurrence, as well as the steep learning curve imposed on residents and fellows in training. This transition was accompanied by adoption of the MICA technique and was further supported by the two-year outcomes reported by Lewis and colleagues.^7,26^

Our institutional MICA experience between 2019 and early 2022 encompassed 264 feet and yielded outcomes consistent with the published literature. In a series of 69 feet with a mean follow-up of 27 months, mean AOFAS scores improved by 36.9 points and hallux valgus angle was corrected by a mean of 30.8°.^20^ In a companion series focused on severe deformities (mean preoperative hallux valgus angle 40.1 degrees, intermetatarsal angle 16.1 degrees), statistically significant corrections were achieved across all radiographic parameters.^27^

The transition to META in April 2022 was underpinned by two complementary rationales. Biomechanically, the chevron configuration carries an intrinsic risk: an overly long lateral wing may lack cortical purchase for bicortical proximal screw fixation, potentially compromising construct stability and predisposing to fracture. The straight transverse cut of META eliminates this risk entirely.^17^

Pedagogically, the linear cut is more consistently reproducible under fluoroscopic guidance by trainees and fellows, reducing radiation exposure and operative time during supervised cases, a consideration of practical importance at a teaching institution. The 12-month outcomes published by Lewis and colleagues for META validated this institutional choice.^17^

Over time, both the MICA and META techniques underwent progressive technical refinement in our practice, particularly with respect to fixation strategy and intraoperative correction maneuvers. One important modification was the management of the medial bony prominence after lateral translation of the first metatarsal head.^28^ Rather than routine contouring with a wedge burr, we progressively adopted resection with the Shannon burr, which provided more efficient handling of the cortical medial prominence and facilitated a more controlled excision.

In parallel, intraoperative assessment of first metatarsal pronation also evolved, with greater emphasis on fluoroscopic evaluation and technical maneuvers aimed at achieving rotational correction before definitive fixation. Together, these refinements improved the reproducibility of deformity correction and contributed to the maturation of both MICA and META as more reliable minimally invasive procedures over time.^4,21,28,29^

The META technique has evolved into a more reproducible minimally invasive procedure because its straight osteotomy is technically simpler to perform and easier to standardize than Chevron-type cuts. However, despite this advantage, fixation remains a critical challenge, particularly with respect to accurate screw trajectory, maintenance of correction, and control of multiplanar alignment.

For this reason, research efforts incorporating innovation and technology have been pursued, including the development of patient-specific 3D-printed instrumentation guides. As described in a recent technical report, these customized guides were designed to assist osteotomy execution and screw placement in fourth-generation minimally invasive hallux valgus surgery, with the aim of improving precision, enhancing safety, shortening operative time, and reducing radiation exposure.^30^

As our osteotomy-based techniques evolved, systematic longitudinal follow-up also guided changes in the treatment of milder deformities. Use of the combined percutaneous exostectomy-Akin (EA) procedure progressively declined after clinical surveillance identified progressive valgus instability in a subset of patients with very mild hallux valgus. This was followed by a marked restriction in its indication, with a significant difference between 2018-2019 and 2022-2023 (p<0.001). Over the same period, mild cases that had previously been treated with EA were increasingly managed with MICA and, more recently, META, although EA was still used selectively in a small number of later cases.

These observations underscore the value of structured postoperative surveillance in assessing the durability of less invasive surgical strategies. A similar process was observed in bilateral simultaneous surgery. The reduction from 45.2% bilateral cases in 2024 to 34.0% in 2025 (p=0.020) followed institutional recognition of a higher fracture rate in bilateral cases, a finding that was subsequently confirmed in a published case-control study.^31^

Age was identified as the main risk factor, independently of bone mineral density, and this finding contributed to the adoption of stricter criteria for simultaneous bilateral surgery from 2025 onward. This sequence also illustrates how high-volume centers with prospective data registries can detect clinically relevant patterns before formal publication, allowing early refinement of surgical indications based on institutional evidence.

### Limitations

This study has several limitations. First, its retrospective design precludes causal inference regarding the effect of each technical transition on outcomes. Changes in practice occurred over time within a dynamic clinical environment and were likely influenced by multiple factors, including surgeon experience, refinement of intraoperative technique, evolving case selection, and broader institutional changes.

Second, the analysis was designed to describe institutional practice evolution rather than to compare patient-level clinical or radiographic outcomes among techniques; those outcome data have been reported separately elsewhere. Third, the experience reflects a single high-volume center with a stable surgical team and a predominantly older patient population, which may differ from the case mix, resources, and training structure of other institutions. Finally, because some treatment indications continued to evolve during the later years of the study, particularly for selected mild deformities, ongoing follow-up remains necessary.

### Interpretation

The present findings show how structured longitudinal surveillance can shape surgical practice in a meaningful way, even outside the setting of randomized trials. Over the study period, each major change in institutional strategy was driven by patterns observed during routine clinical follow-up and then incorporated into subsequent decision-making. In this sense, the study does not present technique evolution as a sequence of isolated technical preferences, but rather as a cumulative process of observation, reassessment, and refinement.

The transitions from RI to MICA, from MICA to META, the progressive restriction of EA as a routine option for mild deformity, and the restriction of simultaneous bilateral procedures all emerged from this iterative process. These data suggest that, in experienced centers with prospective registries, careful monitoring of real-world outcomes can generate practice-relevant evidence that complements the published literature and supports earlier modification of surgical indications.

### Generalizability

Although the specific timeline and technical sequence reported here reflect the experience of a single referral center, the broader lessons are likely relevant to other minimally invasive hallux valgus programs. Centers adopting these procedures may encounter similar phases of transition as they move from earlier-generation techniques toward more reproducible osteotomy-based methods and refine their indications based on observed outcomes.

At the same time, direct extrapolation of our frequencies, thresholds, or institutional preferences should be made with caution, as these may vary according to patient age, bone quality, surgeon experience, training environment, and local follow-up infrastructure. Even so, the central principle appears broadly applicable: prospective outcome monitoring within a high-volume clinical practice can serve as a practical tool for improving patient selection, refining technique, and identifying specific situations in which older procedures may still retain value.

## CONCLUSION

Over eight years and 1,217 operated feet, minimally invasive hallux valgus surgery at our center evolved from Reverdin-Isham to MICA and ultimately to META as the institutional standard. This progression was guided by continuous technical refinement and structured outcome monitoring. META emerged as the preferred technique because it improved reproducibility and simplified execution, while the exostectomy-Akin approach was no longer used routinely and bilateral indications became more restrictive based on observed postoperative outcomes. These findings illustrate how prospective surveillance in a high-volume practice can support evidence-based evolution of minimally invasive hallux valgus surgery.

## Data Availability

The data cannot be made publicly available.
